# Innovations towards sustainable olive crop management: a new dawn by precision agriculture including endo-therapy

**DOI:** 10.3389/fpls.2023.1180632

**Published:** 2023-06-06

**Authors:** Luca Grandi, Michael Oehl, Tommaso Lombardi, Vito Rocco de Michele, Nicolas Schmitt, Dimitri Verweire, Dirk Balmer

**Affiliations:** ^1^ Invaio Sciences, Research & Development Europe, Basel, Switzerland; ^2^ Invaio Sciences, Carovigno, BR, Italy

**Keywords:** *Olea europaea*, precision agriculture (PA), sustainability, endo-therapy, *Xylella fastidiosa*, controlled release, integrated pest and disease management

## Abstract

Olive trees (*Olea europaea* L.) are deeply interwoven with the past, present and future of Mediterranean civilizations, both as essential economical as well as cultural valuables. Olive horticulture constitutes one of the primary agroecosystems in the Mediterranean regions of today. Being inhabitant of ecological niches, *Olea europaea* is prone to a peculiar vulnerability towards climatic and socioeconomical transformations that are briskly reshaping regional and global agroecosystems. Because of climatic changes and the biosafety risks of global agricultural trades, olive plants are highly susceptible to newly emerging diseases and pests, and the traditional olive horticultural crop protection practices are under scrutiny towards reducing their ecological impact. Hence there is an eminent demand for a more nature-positive olive tree crop management. Recent innovations in precision agriculture are raising the prospect for innovative crop protection methods that may provide olive farmers the required agility to respond to present and future agricultural challenges. For instance, endo-therapy, which is the systemic delivery of active ingredients *via* trunk injection, is a technology that holds promise of a true step-change in sustainable olive crop management. Endo-therapy allows reaching vascular diseases inaccessible to foliar treatments and delivers active ingredients in a precise manner with no risks of off-target drifts. Similarly, progresses in biological precision delivery using controlled release of active ingredients based on innovative formulation technologies are showing an exciting potential for more targeted and sustainable crop protection solutions. This review summarizes the latest innovations on both physical and biological precision deliveries in the realm of olive arboriculture in the Mediterranean regions and gives an outlook how these technologies may help orchestrating innovative olive culture practices soon.

## Introduction

The olive tree (*Olea europaea*) is an iconic plant in the Mediterranean regions at present, as well as a symbol that embodies the rich history of splendid human cultures developing in these regions in the past. Recent archeological evidence manifests the use of olive wood and fruits by early *Homo sapiens* on the Atlantic coast of Morocco as early as 100’000 years ago (Marquer et al., 2022). Similarly, sophisticated olive horticultural systems helped thriving ancient cultures in the Central Jordan Valley as early as 7000 years ago (Langgut and Garfinkel, 2022). These palaeoecological findings, as well as the apparent heritage and status of olive tree products in the prevailing Mediterranean diets (Trichopoulou, 2022), clearly corroborate the impetus of this tree on the socioeconomic progress of humankind in this region. Historic olive tree cultivation was key in fuelling long-distance trading and elaborate social contacts, ultimately promoting the wealth of the Mediterranean populations.

Current botanical research trends underline the far-reaching value of olive trees, with a tangible increase in scientific efforts dedicated at olive biology and ecology. For instance, the knowledge of olive genomes is currently rapidly growing, including high-quality genome assemblies of *Olea europaea* subspecies from East Asia (Wang et al., 2022), which start to provide scientists a first fundament of expertise towards genetic resistance and tolerance to abiotic and biotic stresses. At the same time, the biochemical understanding of olive plants is steadily advancing, with state-of-the-art metabolomics approaches that help characterizing olive leave extracts with unprecedented precision down to the fingerprinting of seasonal or variety-specific metabolite fluctuations (Difonzo et al., 2022). Given the known antimicrobial and health benefits of olive metabolites, it does not come as a surprise that a notable focus on current pharmacological research is centred around olive plants, such as the attempt to utilize olive oil-based formulations to combat drug-resistant microbes (Di Pietro et al., 2022). This culminates in the effort to build a public database of chemical compounds originating from olives, which are summarized in the OliveNet™ library (2023). This compound database was recently harnessed for an *in-silico* validation and docking studies of potential interactors with the human Ether-à-go-go-Related Gene (hERG) potassium ion channel (Pitsillou et al., 2022).

While the scientific advances in the olive research field are encouraging, olive agriculture in the Mediterranean basin currently faces significant challenges which threaten its very existence. Not only are there newly emerging difficult-to-control pests and pathogens, but olive cultivation is simultaneously under scrutiny towards replacing traditional crop protection methods with more nature-positive crop management methods. These challenges do not only affect the agricultural sector, but also start to touch other economical branches such as the gastronomic tourism or oleotourism, an emerging concept that start to gain importance in regions heavily invested in olive agriculture such as the province of Córdoba in Spain (Cava Jimenez et al., 2022). Notably, the entire Mediterranean olive product supply chain faces the political and societal pressure to meet the biodiversity and conservation compliance enforced with the European Union New Green Deal and the United Nations Agenda 2030, whilst attempting to be able to competitively provide premium quality produce in a growing international market (Lombardo et al., 2022). Although the political sustainability guidelines offered to olive growers are providing a framework for the transition to new cultural practices, it is evident that disruptive technical innovations for short- and mid-term solutions are scarce. Recent advances in targeted precision endo-therapy, advances in formulation technologies and alternative crop protection products, are raising the hope for a solid portfolio of olive crop management solutions which meet the sustainability standards expected by the public and consumers. This review sets the scene by summarizing emerging pathogens and pests for olive trees, followed by highlighting recent innovations in precision physical delivery of active ingredients, as well as formulation technologies. Finally, an outlook of an integrated olive crop protection management is provided, including remote sensing and predictive science for timely applications, all to reduce the environmental impact of olive cultivation.

## Emerging pests and diseases of olive trees

### Emerging insect pests

**Figure 1:**
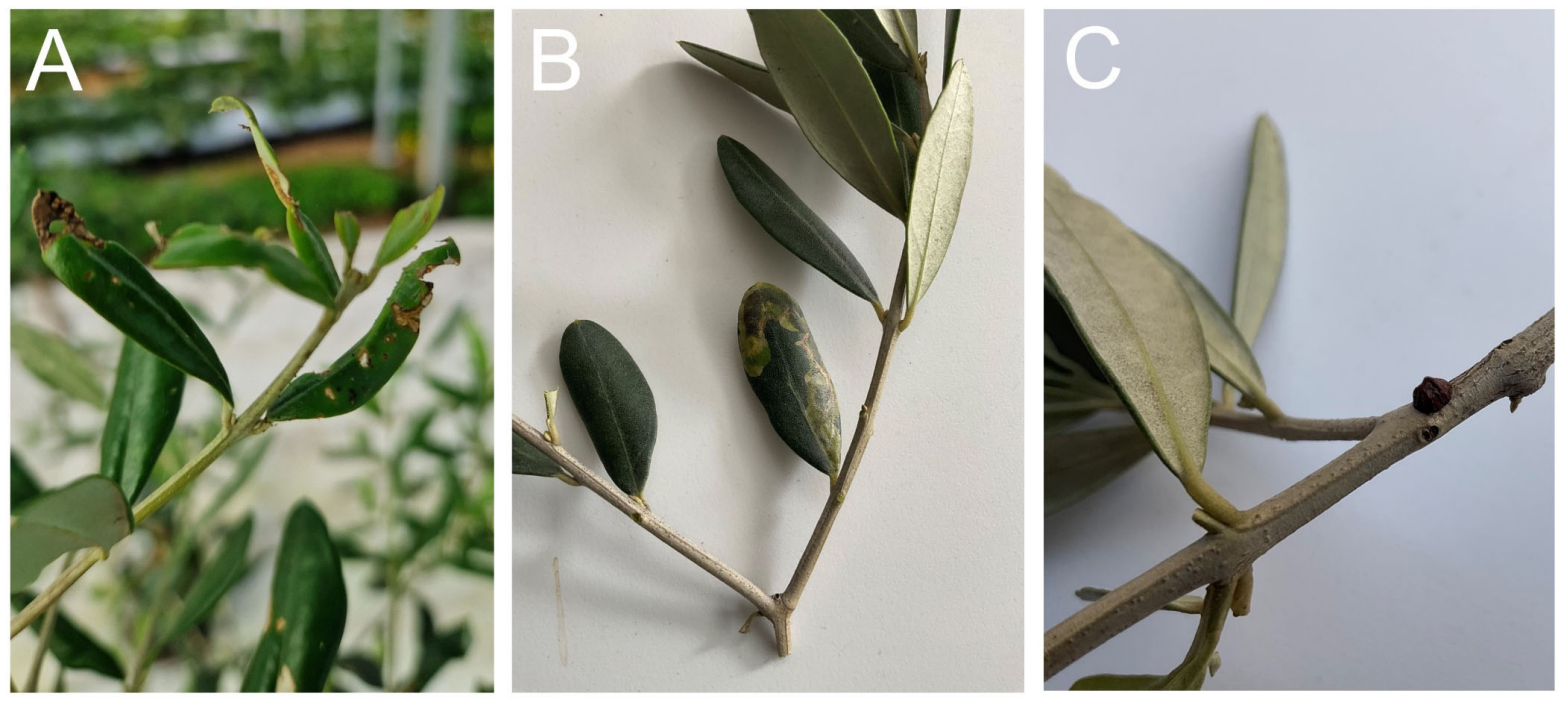
**(A)** Typical foliar herbivory damage caused by the Jasmine moth *Palpita vitrealis* larvae in young olive plants. **(B)** Foliar damage caused by stage IV aged *Prays oleae* (olive moth) larvae. **(C)** The phytophagous parasite *Saissetia oleae* (black scale) on an olive branch. Photos by V. Rocco de Michele.

Being inhabitant to ecological niches, olive trees are exceptionally sensitive to altering pests and pathogen epidemics. Bearing in mind the fact that especially the Mediterranean regions are highly affected by climate-driven amplification of abiotic stresses such as drought and heat, it becomes evident why olive cultivation is highly vulnerable to sudden outbreaks of new diseases, or prolonged exposure to herbivorous insect pests (Caselli and Petacchi, 2021). Today, both insect pests, as well as fungal and bacterial pathogens, pose a concurrent threat to olive growers in the Mediterranean as well as on a global scale. Detrimental exogenous effects triggered by climatic consequences are for instance well documented for the key emerging olive insect pest *Bactrocera oleae* (olive fruit fly), whose larvae feed on drupe pulp and cause massive economical losses (Caselli and Petacchi, 2021). Several studies reviewed in Caselli and Petacchi (2021) show the multifaceted relationship of climate and weather, *B. oleae* parasitoids and predators, as well as temperature-driven population dynamics of the fruit flies, which render predictive pest modelling an arduous task. Similarly, olive moths such as *Prays oleae* are also of growing concern (Ait Mansour et al., 2017), as the gravity of pest attack becomes difficult to foresee with the stronger climatic fluctuations, especially in the coastal areas of Morocco. Other Lepidopteran pests that are persistently advancing are *Euzophera bigella* (quince month), which was noticed on Greek olive trees for the first time in 2011 (Simoglou et al., 2012), and *Palpita vitrealis* (Jasmine moth, **Figure 1**) to which especially younger olive trees in high-density olive cultivation areas are vulnerable (Chaouche et al., 2019). Not only Lepidopteran pests are on the rise, but also Diptera, especially *Dasineura oleae* (olive leaf gall midge). The feeding of *D. oleae* larvae induces foliar gall formation, which affects the leaf physiology and crop quality, up to defoliation phenotypes. Initially a minor pest in olives, recent rises in outbreaks are rendering this insect a significant threat (Tondini and Petacchi, 2019). Advances in detection of *D. oleae* parasitoids are giving hope of means to control *D. oleae* epidemics (Magagnoli et al., 2022), but the economic impact of this pest is likely to grow significantly in the coming years. Meanwhile these emerging pests are also competing for food resources with well-established olive pests, such as the black scale insect *Saissetia oleae* (Tena et al., 2007), which causes stunting, defoliation, dieback, and lack of fruits. Notably, other insects usually seen as secondary pests, such as *Otiorhynchus cribricollis*, are also on the rise in specific areas such as Puglia (field management team Invaio Sciences, Italy; personal communication). Intensification of olive cultivation makes mechanical control (e.g., wrapping olive stems with glass wool) more difficult and favor pest outbreaks especially in younger olive trees. Altogether, the threat these emerging pests constitute to olive growers is considerably amplified by the fact that a growing number of traditional foliar insect control active ingredients will not be available in Mediterranean countries soon.

### Emerging bacterial pathogens

Insects do also play a pivotal role in promoting established as well as emerging microbial pathogens in olive trees. The insect-transmitted gram-negative bacterium *Xylella fastidiosa* is the most notorious microbial pathogen of olive trees in certain regions in the Mediterranean today. Since its original identification as causal agent for the Olive Quick Decline Syndrome (OQDS) in 2013, its pernicious effect on olive horticulture is overwhelming (Trkulja et al., 2022). In the Americas, the principal vector of *X. fastidiosa* is the glassy-winged sharpshooter (*Homalodisca vitripennis*), an invasive insect whose geographic expansion is favored by climatic changes. Species distribution modeling (SDM) studies were recently conducted to generate risk models for future invasion scenarios of this insect in the Mediterranean basin (Rossi and Rasplus, 2023). There, the meadow spittlebug *Philaenus spumarius* acts as key vector for *X. fastidiosa*; its higher prevalence in certain Mediterranean regions has recently also been linked to temperature increases (Farigoule et al., 2022). *Xylella fastidiosa* has a broad host range as commensal and pathogen, with a prevalence in over five hundred plant species, but its shift from a completely commensal to a symptomatic lifestyle in some hosts is not understood. Genetic analysis of both hosts and bacterial subspecies start to shed light into this conundrum (Landa et al., 2022), but recent multistrain genome comparisons of *X. fastidiosa* also revealed previously unknown plasmids (Velasco-Amo et al., 2022), and notably *multiplex* strains from the same sequence type and the same host plant were shown to differ as well in plasmid content and virulence traits (Román-Écija et al., 2022). The genetic plasticity uncovered by these studies may not come as a surprise, as *X. fastidiosa* as a xylem-limited bacteria requires an intricate genetic toolset to be able to thrive in the harsh environment of plant vascular systems with negative pressure, low oxygen and fluctuating nutrient content. The perspectives of combatting *X. fastidiosa* in olive trees are currently modest, given the fact that many traditional bactericidal agrochemicals are not approved for olive cultivation or lack the required systemicity to reach the target *in planta*. Alternative treatments such as fertilizers or antibacterial minerals as well as antimicrobial peptides (Baró et al., 2022) are either not fully explored within existing field programs, or in the case of the latter, still at early research and optimization stages (Morelli et al., 2021; Catalano et al., 2022). Naturally, the endophytic lifestyle of the bacterium limits the efficiency of foliar applications. Notably, the surging incidence of another bacterial pathogen, the gram-negative bacterium *Pseudomonas savastanoi* pv. *savastanoi* causing olive knot disease must be mentioned (Košćak et al., 2023). This pathogen causes hyperplastic growth on olive stem and branches which are often not diagnosed properly, thus underestimating its incidence and making it difficult to quantify the economic losses caused by the bacterium. Although known since more than half a century ago, and being a concern in fruit-producing nurseries worldwide, *P. savastanoi* remains understudied mainly due to the absence of a suitable model system and the complexity of knot-limited life-style in woody tissues (Ramos et al., 2012). It remains elusive which virulence factors *P. savastanoi* requires to infect woody plants compared to herbaceous plants, and which bacterial-derived signals are responsible for tumor growth. Advances in microbiome sequencing technologies and insights into olive xylem microbial communities (Anguita-Maeso et al., 2023) may also help to understand the role of bacterial consortia present in olives in affecting *P. savastanoi* disease severity.

### Emerging fungal pathogens

Limitations for efficient foliar applications can also be anticipated for other vascular diseases including fungi. For instance, the soil-born fungus *Verticillium dahliae* (Verticillium wilt), a hemibiotrophic fungus that is persistent in soils and can grow endophytic in xylem vessels of trees causing nutrient deficiencies, yield reduction and even olive tree death (Montes-Osuna et al., 2020). While *V. dahliae* is not a novel threat for olive orchards, the pathogen also benefits from climatic changes and the intensification of olive cultivation, such as high-density cultures and artificial irrigation, significantly increasing the economic damage of this fungus. For instance, Jiménez-Díaz et al. (2012) showed a correlation of promoted Verticillium wilt infection in high-intensity olive orchards in Andalusia as these were often planted in fields previously cropped with cotton, thus intensively irrigated soils. Together with the fact that efficient strategies to reduce the disease index or control the pathogen dispersal are scarce (López-Moral et al., 2022), it can be assumed that olive growers are going to face *V. dahliae*-caused issues more sharply in the future. Recent biological studies start shedding light into the nutritional imbalances upon *V. dahliae* infection in olives, where potassium deficiency was found to increase the olive wilt disease progress (López-Moral et al., 2022). This study is laying the foundation for further much needed work to help generation integrated pest management programs that may also include dedicated mineral nutrient management. Similarly, recent trials with foliar and root application phosphonate salt copper phosphite (CuPh), which stimulates plant immunity and has antimicrobial properties, show some potential in olive trees (López-Moral et al., 2022). Other strategies for *V. dahliae* control currently being evaluated in olives are the application of beneficial microbes including *Aureobasidium pullulans* AP08 and *Bacillus amyloliquefaciens* PAB-024 (López-Moral et al., 2022). Being a vascular pathogen, more targeted alternatives of foliar or drench application of crop protection products are naturally in the spotlight of Verticillium wilt research. For instance, first studies in the early 2000’s showed protective effects of injected fosetyl-Al and benomyl into young “Nocelleara del Belice” olive trees (Mulè et al., 2002). Notably, both active ingredients were applied with distinct formulations, indicating adaptive protocols are required for optimal compound delivery. The search for alternative fungal control strategies is also on the verge for *Venturia oleaginea* (olive leaf spot), a foliar pathogen which is epidemic in most olive growing regions (Buonaurio et al., 2023). Fungicide resistance, as well as the need for so called eco-friendly solutions including the upcoming expiry of copper products in organic farming in Europe in the near future, are likely to raise the impact of *V. oleaginea* on olive tree cultivation. The enhanced awareness of the above-mentioned diseases in olive cultivation positively fuels current efforts in disease diagnostics across olive orchards in the Mediterranean and on a global scale, which is helping in overall readiness for possible newly emerging diseases of relevance. For instance, the first appearance of the fungus *Pleurostoma richardsiae* in olive trees in Spain was noted lately, while analyzing trees initially diagnosed for *V. dahliae* symptoms (Calvo-Peña et al., 2022). *P. richardsiae* causes twig and branch dieback in olives, with extensive discoloration phenotypes starting at the roots and extending into the trunk when opening the trees. Other fungal species that were recently surprisingly detected on olives are *Dematophora* (ex *Rosellinia*) *necatrix*, and *Emmia lacerate* (Fusco et al., 2022). These were identified in young olive trees suffering lethal wilting in central Italy, while being diagnosed for *X. fastidiosa*. Fusco et al. found bark rotting and xylem discoloration as typical symptoms of D. necatrix, and infections occurred merely at the stems buried during planting. The presence of *E. lacerate*, normally a wood decaying fungus, has not been reported before in olive trees. Although apparently growing symptomless in olives (Fusco et al., 2022), the saprophytic fungus may have a biological role in modifying natural decomposition processes in olive trees which may affect other biotic stresses.

Altogether, olive orchards and their farmers are facing today more than ever significant challenges in terms of biotic and abiotic burdens as well as rising political pressure against traditional crop protection methods. The olive industry as a whole is forced to rapidly adjust towards enhanced sustainability measures across the whole supply chain, hence innovative technologies are required that provide the necessary agility and responsiveness. Aside the quest for novel, more nature-friendly active ingredients, a bright spotlight lays as well on the research of alternative biophysical delivery methods. These technologies bear the potential to reduce the consumption of water, the application rates of active ingredients, and can offer advantages to products that are currently not suitable for field applications due to issues in bioavailability. The following sections summarize recent advances in both biological delivery approaches using innovative formulation methods, as well as progresses in endo-therapy utilizing precision delivery systems into olive trees *via* injections into the vascular system.

## Precision arboriculture using innovative application and formulation technologies: the next wave of olive crop protection solutions?

Precision agriculture, that is the technology of accurate applications of active ingredients both in terms of timing and localization, is notably an area of significant agricultural innovations including in arboriculture. While precision farming will undoubtedly play a pivotal role in the political and public agenda of reducing the environmental impact of agriculture, both technical as well as regulatory aspects are not harmonized to date (Faupel et al., 2023). Nonetheless, significant advances in multiple research fields are constantly advancing the portfolio of available precision farming tools for arboriculture. This encompasses optimized application methods using unmanned aerial vehicles (UAVs) depositing active ingredients using highly adapted formulations, as well as precision applications using controlled-release formulations which improve targetability and efficacy of active ingredients. Although UAVs are currently not considered in olive orchards for spray applications, advances in application assessments in other horticultural fields such as Nanguo pear orchards are starting to build the technical foundation in terms of nozzle and adjuvant selection (Guo et al., 2022). Similarly, application studies for aerial spraying on pine trees are also highlighting the requirement of nozzle selection for an optimal droplet deposition on trees (Yao et al., 2022). While precise deposition of active ingredients using UAVs in olive orchards may become relevant only in the midterm, UAVs/drones are routinely used for multispectral imaging of olive trees which can for instance aid precise detection of the weed *Ecballium elaterium* (Torres-Sánchez et al., 2023). In terms of precision farming in horticulture, a trend is also the use of robotics for tree management including precise monitoring of trunks. For instance, a recent study applied deep learning algorithms to aid a robotic platform equipped with a multisensory device to map and monitor trunks in forest and ornamental settings (da Silva et al., 2022). Robotics can be expected to impact precision horticulture beyond automated fruit collection approaches towards holistic trunk management platforms also in olive cultivation.

Precision farming can also be achieved by harnessing innovative formulation technologies that improve targetability and efficacy of active ingredients and ultimately result in reduced environmental impact (Li et al., 2021). “Smart” drug delivery systems are an integral part in various human medical fields such as cancer therapies (Kalaydina et al., 2018), serving as inspiration of “intelligent” crop protection products that may come with advantages over classical agrochemical formulations. To name a few, these range from lower toxicity to nontarget organisms, increased stability and systemicity, slow-release properties for longer lasting effects, rainfastness and UV protection. Although not many commercial solutions are available to date, the commercial viability of innovative smart release formulations is demonstrated with the successful introduction of Seltima^®^ by BASF in China in 2016. This crop protection product which is targeted at rice blast contains the fungicidal complex III inhibitor pyraclostrobin (CAS 175013-18-0) within an innovative microcapsule formulation. This formulation is releasing the active ingredient upon drying of the microcapsules on the leaf surface (Sampathkumar et al., 2020). Microcapsules falling into the water of the rice paddies remain intact and sink into the bottom mud, where the active ingredient is degraded by microbes, hence significantly minimizing aquatic tox. To date, fundamental scientific knowledge of numerous insecticidal or fungicidal active ingredients encapsulated in various carriers is available (Li et al., 2021). Carriers range from polymers, lipids, clay, metals, and nanocomposite materials, and may also be combined to reach the desired physicochemical features. For instance, encapsulation of the anthelmintic and insecticidal natural product avermectin in cellulose-zein conjugates yielded in a higher UV resistance, better foliar spread and enhanced biological efficacy (Chen et al., 2020). Similarly, recent work by Graily Moradi et al. (2019) on co-encapsulating the insecticides imidacloprid and λ-cyhalothrin in liposomes coated with chitosan show beneficial effects of lipid-based controlled release formulations in *Myzus persicae* bioassays. In some instances, the carrier of nanoformulations itself may play a valid role in antimicrobial activity, as recently shown by Schiavi et al., 2022. Cellulose crystals obtained from waste of olive pruning were shown to inhibit growth of *P. savastanoi*, and are proposed as possible nanocarriers for additional active ingredients for the treatment of olive trees (Schiavi et al., 2022). Altogether, it is evident that advances in controlled-release formulation technologies have the potential to boost the transition towards more nature-friendly crop protection practices as well in olive trees. This may especially be relevant in the light of repurposing natural products or new modalities such as antimicrobial peptides for the control of emerging olive pathogens and pests.

## Endo-therapy in olives: precision agriculture using physical trunk delivery

A particularly intricate precision agriculture method for horticulture consists of directly delivering active ingredients into the plant vascular system by physical injections, or tree endo-therapy. Trunk injections provide significant advances compared to passive delivery mechanisms using soil drench or trunk spray applications for trees (Zamora and Escobar, 2000). For instance, soil drenches are known to be less efficient, less targeted, more impactful for the environment, as well as less rapid in terms of systemic compound distribution as opposed to trunk injections. Traditionally practiced in high-value ornamental plants, fundamental knowledge on commercial trunk injections does exist on both monocot and dicot plants, as well as with manifold injection methods (drill-based, drill-free, with or without pressure) (Archer et al., 2022a). A variety of commercially available trunk injection devices are currently sold, such as solutions from Chemjet (Kerrville, TX, USA), Mauget (Arcadia, CA, USA), Rainbow Ecoscience (Minnetonka, MN, USA), to name a few. The four main categories of injection methods come along each with their advantages and disadvantages, depending also on the crop and active ingredient being applied (Berger and Laurent, 2019; Archer et al., 2022a). For instance, compared to non-drill methods, drill-based devices come with obvious advantages of faster delivery and uptake as they allow the active ingredient to be in contact with a larger xylem area. On the same time, tree damage and wound responses are significantly stronger than with non-drill methods. Comparing low/no pressure devices versus high pressure injections, obvious advantages of higher pressure are reduced application time and larger volumes of active ingredients being administered, whereas low/no pressure devices are known to have less risk of phytotoxic responses as well as lower chances of triggering harmful vessel cavitations (Berger and Laurent, 2019).

**Figure 2:**
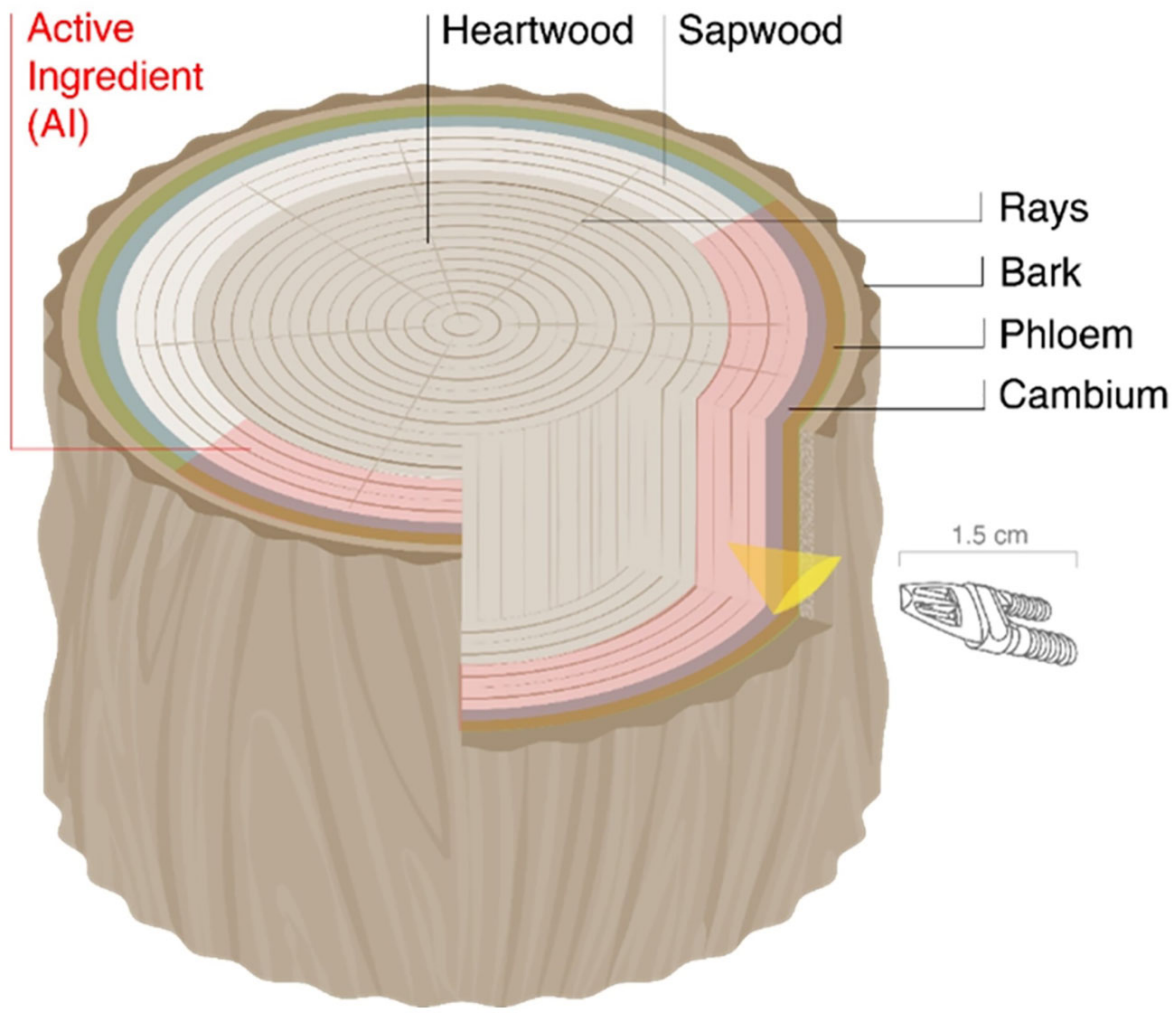
Schematic drawing of the injection head of Invaio Science’s endo-therapy system in context of a tree vascular injection. The drawing is not at scale. The Invaio delivery system (patent pending) specifically infuses an active ingredient (AI) payload into the sapwood, not affecting the heartwood. The adapted design removes the need to drill a whole and to trigger collateral tissue damage that may negatively affect tree health.

Endo-therapy today is commonly used to fight trunk residing diseases and pests as the treatment is directly delivered in the target tissue. Furthermore, this delivery system is also an applicable alternative to conventional foliar spray applications in areas where reduced risk exposure is critical, such as urban public areas. Early extensive tree injection scientific studies go back to the early 20^th^ century, where metal and salt solutions were benchmarked for the control of chestnut blight canker (Rumbold, 1920). In the past 5 years, a significant increase in research papers investigating trunk injections could be observed, not to a surprise correlated with emerging devastating vascular tree diseases that start to negatively impact entire arboricultural business sectors. For instance, Huanglongbing (HLB) disease or citrus greening caused by the phloem-limited gram-negative bacterium *Candidatus* Liberibacter *asiaticus* is inflicting accumulated economic losses over 4.5 billion USD to the Florida citrus business since its emergence in 2005 (Blaustein et al., 2018). Several recent studies investigating the application of systemic antibiotics for the control of HLB using trunk injections in citrus show promising results in terms of disease management (Archer et al., 2022b; Vincent et al., 2022) and demonstrate field efficacy of precision delivery using endo-therapy. These studies also underline the advantages of endo-therapy for cropped trees in terms of sustainability over foliar applications, namely the reduction of required amount of active ingredient, the precision to reach the target disease, and the reduced water consumption as well as unwanted compound drifts. First data on olive tree injection dates from the early 90s, where the uptake and distribution of systemic reporter dyes upon low pressure injection was assessed (Navarro et al., 1992). Similar studies evaluated the injection of ferrous sulfate into olives in order to mitigate iron chlorosis, which is a major issue in olive trees grown in calcareous and alkaline soils (Fernaíndez-Escobar et al., 1993). Treatments in both olives and as well peach trees reduced iron chlorosis symptoms, suggesting an efficient iron application across the two different species using the same injection method and the potential of using trunk injections to combat nutrient deficiencies. Recent work in olive trees shows promising effects of endo-therapy as well in olive cultivation and in the frame of managing *X. fastidiosa*. Injection of the zinc and copper-containing micronutrient fertilizer Dentamet^®^ using innovative endo-therapy with a next generation injection device (**Figure 2**) led to a reduction of *X. fastidiosa* biochemical disease markers (Girelli et al., 2022). This micronutrient-based product was also demonstrated to exert anti *X. fastidiosa* properties *in vitro* at a concentration of 1% (Scortichini et al., 2021). Despite being one of the most devastating and rampant trunk-residing diseases and heavily negatively impacting olive production and the cultural heritage and landscape of Apulia (Italy), an efficient solution to control *X. fastidiosa* has not been found yet. The *in vitro* biological efficacy of metal-based micronutrients does raise the hope for sustainable means of combatting *X. fastidiosa*, but more research is needed to provide olive growers much needed knowledge especially in terms of mid-term and long-term disease management, including controlling the disease vectors today and in the future.

Aside from the clear benefits and promises of endo-therapy in olives, one of its prevailing challenges, especially in anatomically complex woody plants like *O. europeae*, is the even distribution of active ingredients. Trunk injection is a multifaceted process that touches on many different science fields, and although commercially performed for decades in ornamental trees, the scientific community only starts to grasp the entire complexity of the mechanisms (Archer et al., 2022a). Endo-therapy functions within a physiological orchestra of physical, chemical, and biological laws. This is going as far as noting that for a successful precision application using endo-therapy, the “cooperation” of target trees within a given environment is required. Both biotic and abiotic factors need to be considered to understand the distribution of active ingredients and to achieve a successful endo-therapy. Naturally, wood anatomy is a major factor, aside water hydraulics and compartmentalization of decay in given trees (Archer et al., 2022a). Equally importantly, the physicochemical properties of active ingredients, such water solubility, lipophilicity, physiological charge, molecular weight and so one is to be considered in the process of product selection to optimize post-injection absorption and distribution within a tree. Hence, classical agrochemical formulations used for foliar applications may be not suitable or even detrimental for endo-therapy. For example, a study on injecting poorly soluble insecticides or nematicides such as emamectin benzoate in Asian long-horned beetle (ALB)-infested willows highlighted the need of optimal formulations to increase distribution and efficacy (Wang et al., 2020). In some cases, formulation optimization is also required to increase product stability at different temperatures relevant during prolonged agricultural practices during an injection application. Moreover, as in many cases not only a single compound, but mixtures are injected, the compatibility and interaction of active ingredient pairs need to be considered. For instance, Dal Maso et al. (2017) performed an isobolographic analysis of potassium phosphite combined with micronutrients for the control of *Phytophthora cinnamomi* in chestnut using trunk injection. This study corroborates the need for adapted trunk injection formulations that enhance the biological efficacy of injected active ingredients.

**Figure 3:**
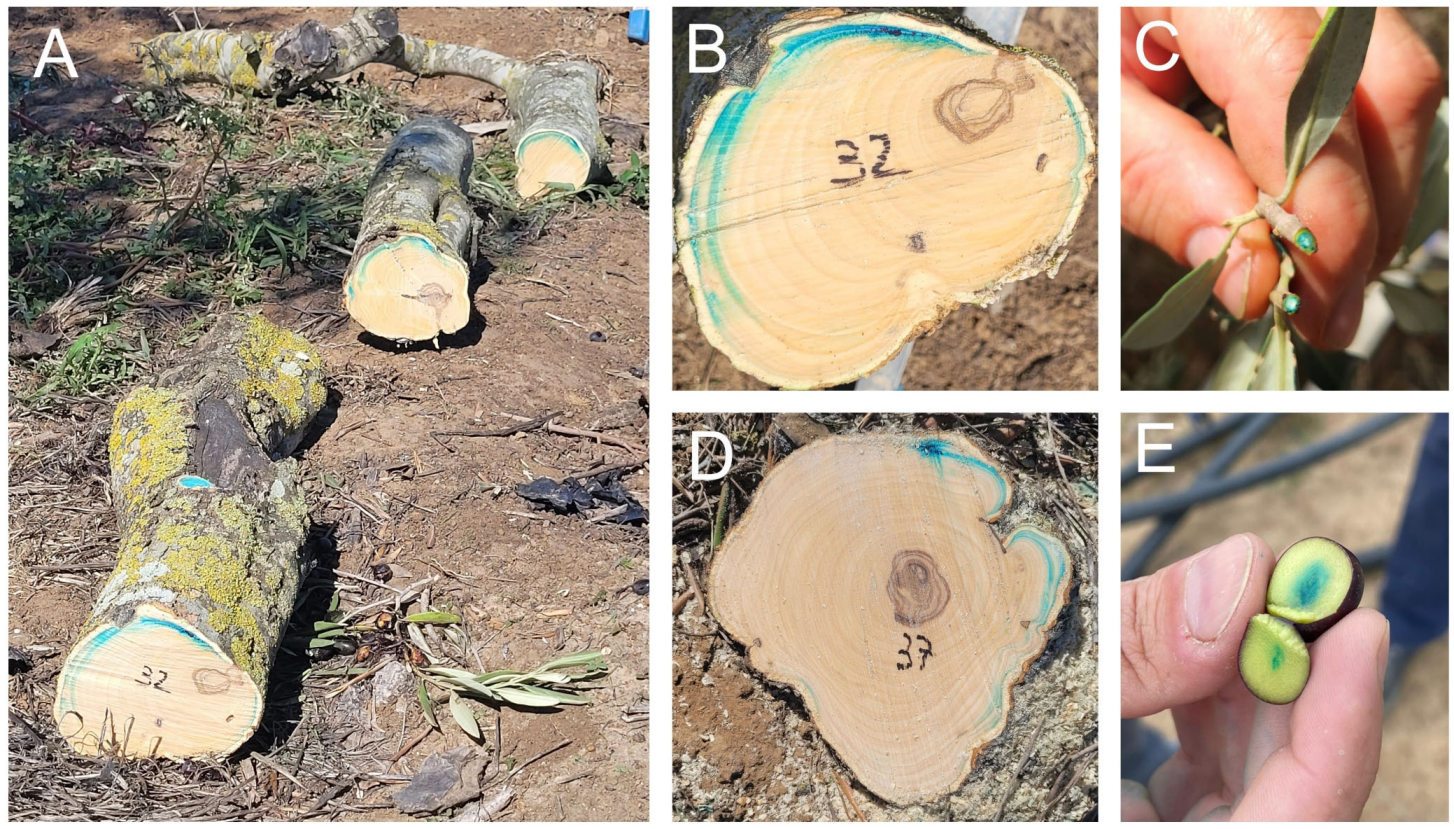
Tracking of systemic active ingredient movement in mature olive trees under field conditions. Erioglaucin disodium salt (CAS 3844-45-9) was injected into a 30 years old Leccino olive tree. A precise distribution within the sapwood could be observed, following an acropetal route to the foliage along the vascular system **(A)**. A radial distribution from the injection point was observed in different trunk shapes **(B, C)**. Long distance movement across the mature tree was demonstrated with the finding of coloured petioles **(D)** and fruits **(E)**, the latter being stained 24 hours post injection.

The above-mentioned scientific questions and challenges around endo-therapy of course also hold true in the case of olive cultivation. The scientific community is only starting to gain a better understanding of olive tree phenology upon endo-therapy, with recent and ongoing research endeavors aiming to elucidate plant responses to vascular injections (Girelli et al., 2022). Across these studies, a prevailing significant challenge is the *in planta* tracking of active ingredients, to monitor the speed and pattern of translocation in the different plant tissues. Injecting synthetic dyes in trees is a prevalent way of assessing vascular biokinetics in trees (Principles and Risks of Trunk Injection for Delivery of Crop Protection Materials, 2021), and this approach is also effective in olive trees (Navarro et al., 1992). For instance, the systemic food dye erioglaucin disodium salt (CAS 3844-45-9) was found to move systemically through the plant when injected into 30 years old Leccino olive trees using Invaio Sciences proprietary targeted infusion (**Figures 3A–E**). Distribution of the injected active ingredient occurs above and below in the injection and following a rapid vertical distribution along the sapwood tissues of the entire tree already at 6 days post injection (**Figure 3A**), with a limited radial distribution (**Figures 3B, C**). Notably, at 24h post injection, the first fruits containing the marker dye were observed (**Figure 3E**). Similarly, petioles of young shoots on the top of the canopy also contained erioglaucin disodium salt, demonstrating a long-distance systemic delivery of the active ingredient in olive trees *via* endo-therapy (**Figure 3D**). Nonetheless, for all different types of injection methods, the complexity of the uttermost even distribution of active ingredients across the whole tree tissue remains a challenge. Early work by Navarro et al. (1992) describes for instance effect of numbers of injection sites, and depth of hole drilled for the low pressure injection, on the overall systemicity of injected active ingredients. Successful endo-therapy relies on a functional sap flow highway, which is influenced by trunk anatomical complexity, physicochemical properties of active ingredients, plant physiology and abiotic as well as biotic factors. As olive trees are pillars within an intricate ecological network reaching from soil microbiomes to meteorological phenomena and carbon sequestration cycles, so are the manipulations of tree vascular biokinetics *via* endo-therapy dependent on a multitude of external factors. Moreover, compared to other tree crops such as citrus or apple, the fundament of many Mediterranean olive cultivation areas are ancient trees bearing a more complex trunk architecture. These aged trees feature a higher degree of compartmentalization by sections of aged and decayed tissue, making endo-therapy in olives a more entangled task. For instance, innovative tree measurement studies from the past have started to lay a foundation of knowledge, such as electrical resistivity data from old versus young olive trees (Al Hagrey, 2006). Thereby, the high resistivity values and therefore more optimal sap flow measured in older trees were hypothesized to be directly linked to harder dry heartwood and cavities in central axes. Younger trees were found to have asymmetric patterns in resistivity, which were correlating with sun and wind directions. Obviously, due to the large differences in trunk anatomy of woody plants (Büntgen et al., 2014), it is an arduous task to transfer endo-therapy knowledge from one tree species to another. For instance, bark tissue or xylem vessel diameter are significantly distinct between olives and citrus. Comparative scientific data elaborating biokinetic differences of molecules across multiple tree species is lacking, and understanding vascular biology of olive trees is in its infancy. Lopez-Bernal et al. (2010) applied a compensation heat pulse method to quantify sap flow and transpiration in olive trees, together with anatomical characterization of xylem tissues, upon exposure of olive trees to different irrigation regimes. They observed an impact of xylem growth and sap flow ratio (nocturnal/diurnal) in deficit-irrigated olives, but no difference in xylem anatomy. Deeper knowledge of the olive vascular system arises from X*. fastidiosa* detection studies using FISH (Fluorescence *In Situ* Hybridization) probes in petioles and branches (Cardinale et al., 2018). Irregular distribution of *X. fastidiosa* within the xylem vessels were detected, as well as signs of horizontal movement of the pathogen, suggesting a complex intercellular network. A more recent study elucidated the sensitivity of a *X. fastidiosa* susceptible and resistant olive cultivar towards xylem cavitation (Sabella et al., 2019). Interestingly, the resistant cultivar seemed less prone to cavitation than the susceptible one, suggesting the presence of more efficient vascular refilling mechanisms in response to the pathogen-triggered loss of hydraulic conductivity. Moreover, a higher number of smaller diameter xylem vessels was observed in cross sections of the resistant cultivar (Sabella et al., 2019), presumably limiting the systemic spread of *X. fastidiosa* compared to the susceptible cultivar with wider xylem vessels.

Additional scientific advances are required to fully understand the biological, chemical, and physical laws orchestrating tree endo-therapy. This comprises advanced insights into the anatomy and biology of olive vascular systems, as well as the behavior of specific active ingredients in the context of vascular transport highways within olives. For olives in particular, but also other crops, significant gaps in both aspects need to be addressed in order to fully harness the potential of endo-therapy. From the farmers point view, a successful endo-therapy relies on the choice of the most efficient device and when to use it with which product. Aside the most common pressured injection system, alternatives such as a drill-free methods of pressure-free infusion are evaluated in some trees such as the English oak *Quercus robur* (Montecchio, 2013). This system uses a specifically designed blade that is claimed to lead to a Venturi effect by separating woody fibers while reducing cross sections of the vessels, thus increasing sap velocity. This work, amongst other ongoing academic and industrial efforts, shows the available innovation space in the technology of tree endo-therapy. With a promising fundament of emerging research concepts, endo-therapy has all the attributes to provide olive growers the much-needed innovation to adapt to current challenges in olive horticulture.

## Sustainable integrated pathogen and pest management in future olive orchards: an outlook

Olive orchards are deep-rooted in the scenery of agricultural regions in the Mediterranean basin, and they have formed over many centuries the now so typical landscapes associated with the cultural and agricultural wealth of Mediterranean countries. Mediterranean olive growers today are facing dynamic commercial challenges triggered by climate change, pathogen and pest shifts, declining workforce, and competition with high-density olive cultivations in the global market to name a few. Looking at the past two decades, olive production from the Mediterranean basin underwent an upward trend, although fluctuations due to biennial or alternative fruit bearings are apparent (Fraga et al., 2020), thus there is an evident growth potential. With sustainability and reduced environmental impact in the socioeconomic agenda of many Mediterranean countries, modernization of olive cultivation is an inevitable requisite to maintain high standards of olive production and to fuel agroeconomic growth. Notably, modernization of fruit orchard cultivation is currently a dynamic research field with academic and industrial collaborations between diverse branches of science from agronomy to engineering, and despite being not in the very spotlight, olive cultivation is starting to benefit from the achievements in other fruit crops such as apple cultivation. The “orchard of the future” concept has been described recently by partners from the Washington State Department of Agriculture, the Dutch government, and universities from California and the Netherlands (Technology solutions for future proofing fruit cultivation, 2021) as umbrella to combine technologies for a more efficient, smart, and sustainable fruit cultivation toolbox. This toolbox may soon enable a truly integrated pest management system (IPM) in orchards, thus a thoughtfully combined approach of biological, chemical, physical, and cultural management strategies with a focus on minimizing risks on human health and environmental impact.

**Figure 4:**
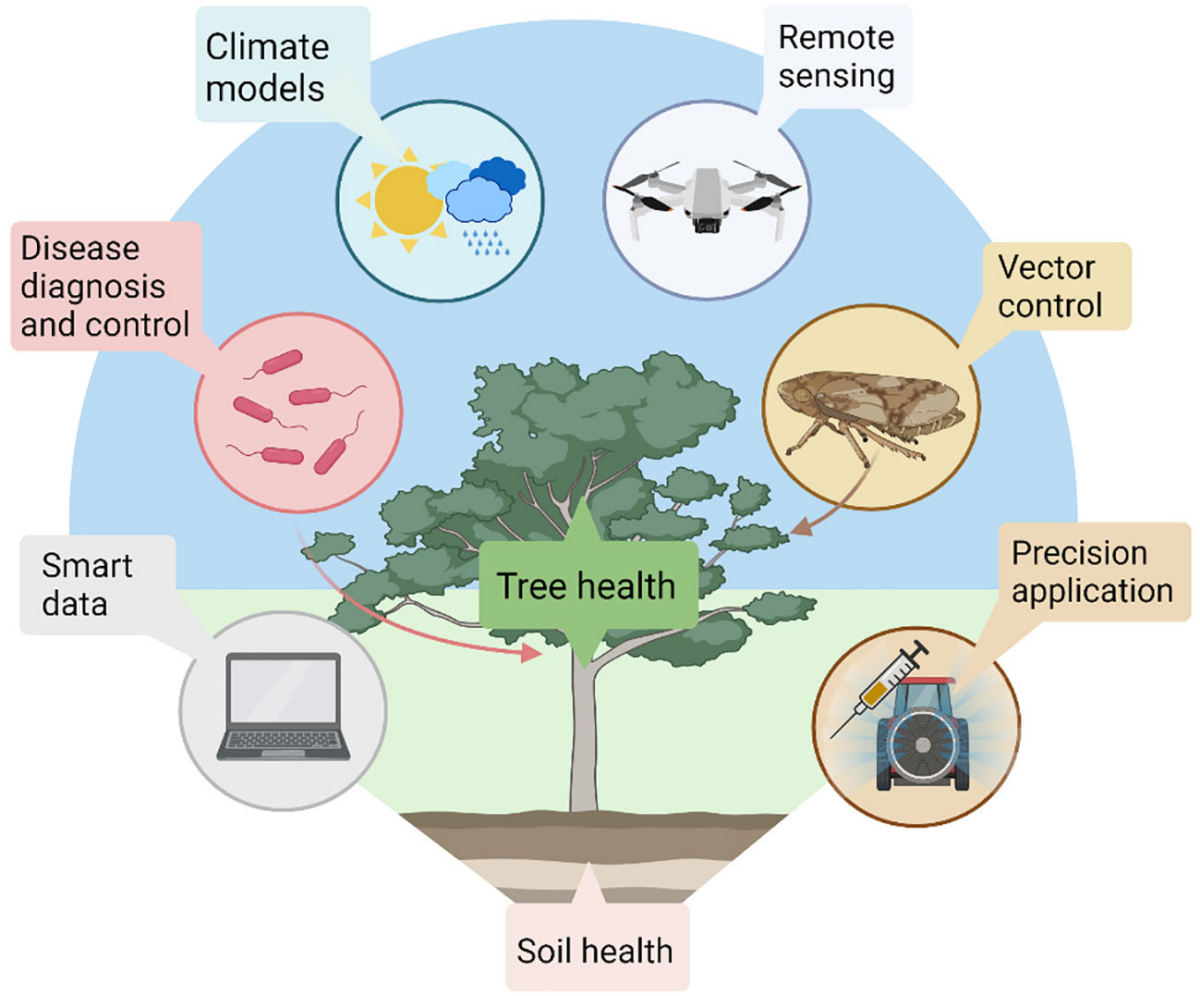
Proposed olive cultivation in the context of orchards of the future. With tree health in the centre, olive cultivation may be efficiently managed with a combination of innovations ranging from automated soil mapping, disease diagnostics and alternative control methods, climate forecasting, remote sensing of biophysical parameters, thoughtful vector control and precision applications using controlled release formulations and endo-therapy. These technologies may be managed in real time by growers using smart data systems. Created with BioRender.com.

Olive orchards of the future may also readily embrace aspects of combinations of intelligent fruit farming innovations from other crops (**Figure 4**). These innovations are likely going to be pivotal in preserving the heritage of olive cultivation in some Mediterranean regions heavily affected by *X. fastidiosa*, as cutting down infected olive trees and replacing them with more tolerant varieties such as Leccino or Favolosa is not a sustainable solution to perpetuate olive cultivation on a long term. Automated tree and soil health assessments may provide the fundament to be merged with technologies addressing disease diagnostics and control, weather predictions, remote sensing, next generation pathogen vector control, innovative precision applications and a smart data management system that offers the growers an edge in data-driven decision making. For instance, soil health monitoring in almond orchards in the region of Lleida (Catalonia, Spain) using galvanic soil sensors have shown to be useful in detecting soil constraints within orchards affected by previous parcelling (Uribeetxebarria et al., 2018). Such automated soil mapping, when combined with *ad hoc* soil sampling for sensor calibration, are providing important soil data including physical properties, macro- and micronutrients and complex data models such as planta available water in olive orchards as well. Aside soil and tree health, timely and automated disease and pest detection will play a pivotal part in the olive orchard of the future. For example, promising results of early detection of *Xylella fastidiosa* using UV multispectral imaging was recently generated in a study conducted in Apulia (Castrignanò et al., 2020). Although it may be challenging to distinguish pathogen-triggered symptoms from abiotic stress in some cases, if combined with advanced deep learning algorithms UAV-produced imaging data does have the potential to aide detection and classification of olive foliar diseases (Ksibi et al., 2022). Concomitant application of climate models to disease and pest forecasts will likely also be an important aspect in the olive orchard of the future. Being inhabitant to ecological niches with narrow climatic buffers, timely responding to climatic stress is crucial in olive orchards (Fraga et al., 2020). Profound understanding of weather conditions is also influencing vector control and precision application strategies. Another highly promising technology supporting olive growers is remote sensing, that is the collection of agricultural information without direct interaction with the specific trees or locations. Remote sensing has a solid history within olive orchard management and is feeding since the early 2000´s into on-farm mapping of data used for pruning, fertilization, irrigation and harvesting to name a few (Messina and Modica, 2022). A variety of studies on remote sensing performed by either satellites, aircrafts or UAVs are providing an excellent foundation to understand the potential and limitations of this technology in olive orchards. Detection and phenotyping of biophysical parameters is a well progressed area, and various algorithms to calculate different vegetation indexes in olives are established (Messina and Modica, 2022). Recent technical advances in image accuracy (spatial, temporal, spectral) are certainly pushing this technology further for an efficient use in orchards of the future. Even though the potential of remote sensing for olive orchards is apparent, the full commercial utilization is hindered due to absence of standards in data collection and processing, and the synergized dissemination and application of remote sensing data across the fragmented olive cultivation areas from traditional and marginal to high-density intensive olive growing farms.

Within an IPM system that can be envisaged for the olive orchard of the future, next generation vector control is likely going to play an important role towards more sustainability in olive farming. The example of *X. fastidiosa* epidemiology corroborates that only a thoughtful combination of different vector controlling strategies may be successful, due to the biological complexity of the insect hosts. For instance, foliar insecticidal sprays or insecticide trunk injections can be used to control one or multiple hosts, but may rapidly promote resistance, thus alternative methods are required. In ornamental sweet olive trees *Osmanthus fragrans*, the injection of several insecticides using a now-pressure device was addressed in terms of uptake and biological efficacy against the nettle caterpillar *Latoia lepida* (Huang et al., 2016). Thereby, it was found that emamectin benzoate had a slower uptake and distribution in the sweet olive trees compared to imidacloprid and abamectin, but the overall had a longer duration and higher potency of insecticidal activity. These results are encouraging in terms of the potential of using insecticide injections in olives, but also leave the question open whether the spread of microbial diseases transmitted by the insects can be contained. Interestingly, recent studies point out the possibility of habitat manipulation to control *Philaneus spumaris* management (Morente et al., 2022). Thereby, utilizing *Anthricus cerefolium* plants as ground cover may help to reduce *P. spumaris* populations, as these plants promote oviposition but are detrimental to the first nymphal instars. Precision delivery of insecticidal active ingredients *via* trunk endo-therapy into olive trees does offer an additional promising vector control method that would massively reduce the volume of applied crop protection products. Exact numbers are depending on specific products, crops, and application methods, but historic studies of foliar applications estimate in general a 99.9% loss of pesticides into the environment with less than 0.1% of active ingredients actually coming in contact with the target pests (Pimentel, 1995). Whilst injection of insect-controlling active ingredients into olives may even result in repelling effects that would significantly interfere with uptake and transmission of bacterial diseases, it is noteworthy that the successful transmission of *X. fastidiosa* by spittlebugs requires only a few minutes, thus may happen rapidly even in the presence of a repellent (Cornara et al., 2020). Finally, *X. fastidiosa* vectors can thrive on many other plant hosts present in proximity to olive trees, thus generating a free-choice situation that may significantly impact the efficacy of insecticides (Lago et al., 2022). With this in mind, targeted delivery of crop protection products using innovative endo-therapy can play an important part in IPM of olive orchards. It may allow a both preventive as well as curative solution that can act synergistically within other IPM methods, while clearly following sustainability guidelines by significantly reducing environmental risks.

Altogether, the future of olive cultivation looks bright with contemporary innovations in precision delivery technologies, remote sensing, and progress in digital tools. Combining all these with a smart data system will allow for more efficient and effective pest and disease management, resulting in healthier and more productive and profitable olive orchards. The integration of the above-mentioned technologies into olive arboriculture is critical to achieving sustainable IPM, which aims to reduce the overall use of traditional synthetic pesticides whilst effectively managing pests and diseases. This integrated approach shall lead to healthier and more productive olive orchards, ensuring a competitive and sustainable future for olive cultivation in the Mediterranean basin.

## Summary

Aside the substantial challenges olive farmers are facing, olive cultivation is also an exciting research field that is on the forefront of cutting-edge agricultural innovations from precision endo-therapy to remote sensing. The new waves of technologies will undoubtedly be fuelling the reinforcement of Mediterranean olive sector and support its further growth. Nonetheless, olive research would benefit from prioritization and a focus on synergism between the public and private sectors, as well as collaborative efforts in between the fragmented olive growing regions. With all the clear limitations of olive cultivation modernization, such as difficulties of mechanization, new innovative tools such as precision endo-therapy are going to give olive farmers relevant solutions to combat biotic and abiotic stresses. Endo-therapy was shown to reach systemic tissues of mature olive trees while using a limited volume of injected ingredients This highlights new opportunities in treating vascular and foliar diseases and pests, as well as efficient administration of micronutrients and biostimulants for general tree health, whilst significantly reducing off-target environmental impact. Combined with other innovations and embedded within a smart data umbrella, endo-therapy bears disruptive potential for olive orchards, which can be a pioneering example of integrated pest management in arboriculture in general.

## Author contributions

All authors listed have made a substantial, direct, and intellectual contribution to the work and approved it for publication.

## References

[B1] Ait Mansour A. A. Ouanaimi F. Chemseddine M. Boumezzough A. (2017). Study of the flight dynamics of *Prays oleae* (Lepidoptera: yponomeutidae) using sexual trapping in olive orchards of essaouira region, Morocco. J. entomology zoology Stud. 5, 943–952.

[B2] Al Hagrey S. A. (2006). Electrical resistivity imaging of tree trunks. Near Surface Geophysics 4, 179–187. doi: 10.3997/1873-0604.2005043

[B3] Anguita-Maeso M. Navas-Cortés J. A. Landa B. B. (2023). Insights into the methodological, biotic and abiotic factors influencing the characterization of xylem-inhabiting microbial communities of olive trees. Plants 12, 912. doi: 10.3390/plants12040912 36840260 PMC9967459

[B4] Archer L. Crane J. H. Albrecht U. (2022a). Trunk injection as a tool to deliver plant protection materia–s - an overview of basic principles and practical considerations. Horticulturae 8 (6), 552. doi: 10.3390/horticulturae8060552

[B5] Archer L. Kunwar S. Alferez F. Batuman O. Albrecht U. (2022b). Trunk injection of oxytetracycline for huanglongbing management in mature grapefruit and sweet orange trees. Phytopathology. doi: 10.1094/PHYTO-09-22-0330-R 36474420

[B6] Baró A. Saldarelli P. Saponari M. Montesinos E. Montesinos L. (2022). Nicotiana benthamiana as a model plant host for xylella fastidiosa: control of infections by transient expression and endotherapy with a bifunctional peptide. Front. Plant Sci. 13. doi: 10.3389/fpls.2022.1061463 PMC9752042 36531347

[B7] Berger C. Laurent F. (2019). Trunk injection of plant protection products to protect trees from pests and diseases. Crop Prot. 124, 104831. doi: 10.1016/j.cropro.2019.05.025

[B8] Blaustein R. A. Lorca G. L. Teplitski M. (2018). Challenges for managing *Candidatus* liberibacter spp. (Huanglongbing disease pathogen): current control measures and future directions. Phytopathology 108, 424–435. doi: 10.1094/PHYTO-07-17-0260-RVW 28990481

[B9] Büntgen U. Psomas A. Schweingruber F. H. (2014). Introducing wood anatomical and dendrochronological aspects of herbaceous plants: applications of the xylem database to vegetation science. J. Veg Sci. 25, 967–977. doi: 10.1111/jvs.12165

[B10] Buonaurio R. Almadi L. Famiani F. Moretti C. Agosteo G. E. Schena L. (2023). Olive leaf spot caused by *Venturia oleaginea*: an updated review. Front. Plant Sci. 13. doi: 10.3389/fpls.2022.1061136 PMC9868462 36699830

[B11] Calvo-Peña C. Burgos M. Diez-Galán A. Ibáñez A. Coque J. J. R. Cobos R. (2022). First report of *Pleurostoma richardsiae* associated with twig and branch dieback of olive trees in Spain. Plant Dis. 106, 1981. doi: 10.1094/PDIS-10-21-2127-PDN

[B12] Cardinale M. Luvisi A. Meyer J. B. Sabella E. De Bellis L. Cruz A. C.. (2018). Specific fluorescence in situ hybridization (FISH) test to highlight colonization of xylem vessels by xylella fastidiosa in naturally infected olive trees (Olea europaea l.). Front. Plant Sci. 9. doi: 10.3389/fpls.2018.00431 PMC5897508 29681910

[B13] Caselli A. Petacchi R. (2021). Climate change and major pests of Mediterranean olive orchards: are we ready to face the global heating? Insects 12, 802. doi: 10.3390/insects12090802 34564243 PMC8472548

[B14] Castrignanò A. Belmonte A. Antelmi I. Quarto R. Quarto F. Shaddad S.. (2020). Semi-automatic method for early detection of *Xylella fastidiosa* in olive trees using UAV multispectral imagery and geostatistical-discriminant analysis. Remote Sens. 13, 14. doi: 10.3390/rs13010014

[B15] Catalano A. Ceramella J. Iacopetta D. Mariconda A. Scali E. Bonomo M. G.. (2022). Thidiazuron: new trends and future perspectives to fight *Xylella fastidiosa* in olive trees. Antibiotics (Basel) 11 (7), 947. doi: 10.3390/antibiotics11070947 35884201 PMC9312276

[B16] Cava Jimenez J. A. Millán Vázquez de la Torre M. G. Dancausa Millán M. G. (2022). Factors that characterize oleotourists in the province of córdoba. PLoS One 17, e0276631. doi: 10.1371/journal.pone.0276631 36327197 PMC9632825

[B17] Chaouche S. T. Bengouga K. Fadlaoui H. (2019). The first detection of the olive leaf *moth palpita vitrealis* (Rossi) (Lepidoptera: pyralidae) as a serious pest in biskra province (Algeria). Bull. OEPP 49, 593–596. doi: 10.1111/epp.12607

[B18] Chen L. Zhou H. Hao L. Li Z. Xu H. Chen H.. (2020). Dialdehyde carboxymethyl cellulose-zein conjugate as water-based nanocarrier for improving the efficacy of pesticides. Ind. Crops Products 150, 112358. doi: 10.1016/j.indcrop.2020.112358

[B19] Cornara D. Marra M. Morente M. Garzo E. Moreno A. Saponari M.. (2020). Feeding behavior in relation to spittlebug transmission of *Xylella fastidiosa* . J. Pest Sci. 93, 1197–1213. doi: 10.1007/s10340-020-01236-4

[B20] da Silva D. Q. dos Santos F. N. Filipe V. Sousa A. J. Oliveira P. M. (2022). Edge AI-based tree trunk detection for forestry monitoring robotics. Robotics 11, 136. doi: 10.3390/robotics11060136

[B21] Difonzo G. Crescenzi M. A. Piacente S. Altamura G. Caponio F. Montoro P. (2022). Metabolomics approach to characterize green olive leaf extracts classified based on variety and season. Plants 11, 3321. doi: 10.3390/plants11233321 36501360 PMC9735528

[B22] Di Pietro M. Filardo S. Mattioli R. Francioso A. Raponi G. Mosca L. (2022). Extra Virgin Olive Oil-Based Green Formulations With Promising Antimicrobial Activity Against Drug-Resistant Isolates. Front. Pharmacol. 13, 885735. doi: 10.3389/fphar.2022.885735 35548334 PMC9082028

[B23] Farigoule P. Chartois M. Mesmin X. Lambert M. Rossi J.-P. Rasplus J.-Y.. (2022). Vectors as sentinels: rising temperatures increase the risk of *Xylella fastidiosa* outbreaks. Biology 11, 1299. doi: 10.3390/biology11091299 36138778 PMC9495951

[B24] Faupel M. Von Blanckenhagen F. Lückmann J. Ruf D. Wiedemann G. Ludwigs J. (2023). Precision farming and environmental pesticide regulation in the EU–how does it fit together? Integr. Envir Assess. Manag 19, 17–23. doi: 10.1002/ieam.4626 35502684

[B25] Fernaíndez-Escobar R. Barranco D. Benlloch M. (1993). Overcoming iron chlorosis in olive and peach trees using a low-pressure trunk-injection method. HortSci 28, 192–194. doi: 10.21273/HORTSCI.28.3.192

[B26] Fraga H. Moriondo M. Leolini L. Santos J. A. (2020). Mediterranean Olive orchards under climate change: a review of future impacts and adaptation strategies. Agronomy 11, 56. doi: 10.3390/agronomy11010056

[B27] Fusco V. Pasciuta V. Lumia V. Matere A. Battaglia V. Bertinelli G.. (2022). Root and stem rot, and wilting of olive tree caused by *Dematophora necatrix* and associated with *Emmacerateata* in central Italy. Eur. J. Plant Pathol. 163 (1), 71–96. doi: 10.1007/s10658-022-02458-1 35095205 PMC8783780

[B28] Girelli C. R. Hussain M. Verweire D. Oehl M. C. Massana-Codina J. Avendaño M. S.. (2022). Agro-active endo-therapy treated *Xylella fastidiosa* subsp. *pauca*-infected olive trees assessed by the first 1H-NMR-based metabolomic study. Sci. Rep. 12, 5973. doi: 10.1038/s41598-022-09687-8 35396514 PMC8993878

[B29] Graily Moradi F. Hejazi M. J. Hamishehkar H. Enayati A. A. (2019). Co-Encapsulation of imidacloprid and lambda-cyhalothrin using biocompatible nanocarriers: characterization and application. Ecotoxicology Environ. Saf. 175, 155–163. doi: 10.1016/j.ecoenv.2019.02.092 30897414

[B30] Guo S. Yao W. Xu T. Ma H. Sun M. Chen C.. (2022). Assessing the application of spot spray in nanguo pear orchards: effect of nozzle type, spray volume rate and adjuvant. Pest Manage. Sci. 78, 3564–3575. doi: 10.1002/ps.6999 35598076

[B31] Huang J. Zhang J. Li Y. Li J. Shi X.-H. (2016). Evaluation of the effectiveness of insecticide trunk injections for control of latoia lepida (Cramer) in the sweet olive tree osmanthus fragrans. PeerJ 4, e2480. doi: 10.7717/peerj.2480 27688974 PMC5036080

[B32] Jiménez-Díaz R. M. Cirulli M. Bubici G. Del Mar Jiménez-Gasco M. Antoniou P. P. Tjamos E. C. (2012). Verticillium wilt, a major threat to olive production: current status and future prospects for its management. Plant Dis. 96, 304–329. doi: 10.1094/PDIS-06-11-0496 30727142

[B33] Kalaydina R. V. Bajwa K. Qorri B. Decarlo A. Szewczuk M. R. (2018). Recent advances in "smart" delivery systems for extended drug release in cancer therapy. Int. J. nanomedicine 13, 4727–4745. doi: 10.2147/IJN.S168053 30154657 PMC6108334

[B34] Košćak L. Lamovšek J. Đermić E. Tegli S. Gruntar I. Godena S. (2023). Identification and characterisation of *Pseudomonas savastanoi* pv. *savastanoi* as the causal agent of olive knot disease in Croatian, Slovenian and Portuguese olive (*Olea europaea* l.) orchards. Plants 12, 307. doi: 10.3390/plants12020307 36679019 PMC9865541

[B35] Ksibi A. Ayadi M. Soufiene B. O. Jamjoom M. M. Ullah Z. (2022). MobiRes-net: a hybrid deep learning model for detecting and classifying olive leaf diseases. Appl. Sci. 12, 10278. doi: 10.3390/app122010278

[B36] Lago C. Cornara D. Minutillo S. A. Moreno A. Fereres A. (2022). Feeding behaviour and mortality of *Philaenus spumarius* exposed to insecticides and their impact on *Xylella fastidiosa* transmission. Pest Manage. Sci. 78, 4841–4849. doi: 10.1002/ps.7105 PMC9804339 35908181

[B37] Landa B. B. Saponari M. Feitosa-Junior O. R. Giampetruzzi A. Vieira F. J. D. Mor E.. (2022). *Xylella fastidiosa* ‘s relationships: the bacterium, the host plants, and the plant microbiome. New Phytol. 234, 1598–1605. doi: 10.1111/nph.18089 35279849

[B38] Langgut D. Garfinkel Y. (2022). 7000-year-old evidence of fruit tree cultivation in the Jordan valley, Israel. Sci. Rep. 12, 7463. doi: 10.1038/s41598-022-10743-6 35523827 PMC9076912

[B39] Li N. Sun C. Jiang J. Wang A. Wang C. Shen Y.. (2021). Advances in controlled-release pesticide formulations with improved efficacy and targetability. J. Agric. Food Chem. 69, 12579–12597. doi: 10.1021/acs.jafc.0c05431 34672558

[B40] Lombardo L. Farolfi C. Tombesi S. Novelli E. Capri E. (2022). Development of a sustainability technical guide for the Italian olive oil supply chain. Sci. Total Environ. 820, 153332. doi: 10.1016/j.scitotenv.2022.153332 35074385

[B41] Lopez-Bernal A. Alcantara E. Testi L. Villalobos F. J. (2010). Spatial sap flow and xylem anatomical characteristics in olive trees under different irrigation regimes. Tree Physiol. 30, 1536–1544. doi: 10.1093/treephys/tpq095 21081652

[B42] López-Moral A. Agustí-Brisach C. Ruiz-Blancas C. Antón-Domínguez B. I. Alcántara E. Trapero A. (2022). Elucidating the effect of nutritional imbalances of n and K on the infection of *Verticillium dahliae* in olive. JoF 8, 139. doi: 10.3390/jof8020139 35205893 PMC8880142

[B43] Magagnoli S. Tondini E. Ratti C. Burgio G. Petacchi R. (2022). A new PCR based molecular method for early and precise quantification of parasitization in the emerging olive pest *Dasineura oleae* . Pest Manage. Sci. 78 (5), 1842–1849. doi: 10.1002/ps.6802 PMC9305150 35060274

[B44] Marquer L. Otto T. Arous E. B. Stoetzel E. Campmas E. Zazzo A.. (2022). The first use of olives in Africa around 100,000 years ago. Nat. Plants 8, 204–208. doi: 10.1038/s41477-022-01109-x 35318448

[B45] Messina G. Modica G. (2022). The role of remote sensing in olive growing farm management: a research outlook from 2000 to the present in the framework of precision agriculture applications. Remote Sens. 14, 5951. doi: 10.3390/rs14235951

[B46] Montecchio L. (2013). A venturi effect can help cure our trees. JoVE, 51199. doi: 10.3791/51199 24121874 PMC3938323

[B47] Montes-Osuna N. Mercado-Blanco J. (2020). Verticillium Wilt of Olive and Its Control: What Did We Learn during the Last Decade? Plants 9, 735. doi: 10.3390/plants9060735 32545292 PMC7356185

[B48] Morelli M. García-Madero J. M. Jos Á. Saldarelli P. Dongiovanni C. Kovacova M.. (2021). Xylella fastidiosa in olive: a review of control attempts and current management. Microorganisms 9, 1771. doi: 10.3390/microorganisms9081771 34442850 PMC8397937

[B49] Morente M. Ramírez M. Lago C. de las Heras-Bravo D. Benito A. Moreno A.. (2022). Habitat manipulation for sustainable management of *Philaenus spumarius*, the main vector of *Xylella fastidiosa* in Europe. Pest Manag Sci. 78, 4183–4194. doi: 10.1002/ps.7036 35690910

[B50] Mulè R. Fodale A. S. Tucci A. (2002). Control of olive verticillium wilt by trunk injection with different doses of fosetyl-al and benomyl. Acta Hortic. 587, 761–764. doi: 10.17660/ActaHortic.2002.586.164

[B51] Navarro C. Fernaíndez-Escobar R. Benlloch M. (1992). A low-pressure, trunk-injection method for introducing chemical formulations into olive trees. jashs 117, 357–360. doi: 10.21273/JASHS.117.2.357

[B52] OliveNet™ library McCord research. Available at: https://mccordresearch.com.au/library/ (Accessed February 27, 2023).

[B53] Pimentel D. (1995). Amounts of pesticides reaching target pests: environmental impacts and ethics. J. Agric. Environ. Ethics 8, 17–29. doi: 10.1007/BF02286399

[B54] Pitsillou E. Liang J. J. Beh R. C. Prestedge J. Catak S. Hung A.. (2022). Identification of novel bioactive compounds from *Olea europaea* by evaluation of chemical compounds in the OliveNet™ library: in silico bioactivity and molecular modelling, and *in vitro* validation of hERG activity. Comput. Biol. Med. 142, 105247. doi: 10.1016/j.compbiomed.2022.105247 35077933

[B55] Principles and Risks of Trunk Injection for Delivery of Crop Protection Materials (2021) Citrus industry magazine. Available at: https://citrusindustry.net/2021/05/17/principles-and-risks-of-trunk-injection-for-delivery-of-crop-protection-materials/ (Accessed April 10, 2023).

[B56] Ramos C. Matas I. M. Bardaji L. Aragón I. M. Murillo J. (2012). Pseudomonas savastanoi pv. savastanoi. Mol. Plant Pathol. 13, 998–1009. doi: 10.1111/j.1364-3703.2012.00816.x 22805238 PMC6638699

[B57] Román-Écija M. Navas-Cortés J. A. Velasco-Amo M. D. P. Arias-Giraldo L. F. Gomez L. M. de la Fuente L.. (2022). Two *Xylella fastidiosa* subsp. multiplex strains isolated from almond in Spain differ in plasmid content and virulence traits. Phytopathology. doi: 10.1094/PHYTO-06-22-0234-R 36576402

[B58] Rossi J.-P. Rasplus J.-Y. (2023). Climate change and the potential distribution of the glassy-winged sharpshooter (*Homalodisca vitripennis*), an insect vector of *Xylella fastidiosa* . Sci. Total Environ. 860, 160375. doi: 10.1016/j.scitotenv.2022.160375 36423847

[B59] Rumbold C. (1920). Effect on chestnuts of substances injected into their trunks. Am. J. Bot. 7 (2), 45–56. doi: 10.2307/2435039

[B60] Sabella E. Aprile A. Genga A. Siciliano T. Nutricati E. Nicolì F.. (2019). Xylem cavitation susceptibility and refilling mechanisms in olive trees infected by xylella fastidiosa. Sci. Rep. 9, 9602. doi: 10.1038/s41598-019-46092-0 31270378 PMC6610111

[B61] Sampathkumar K. Tan K. X. Loo S. C. J. (2020). Developing nano-delivery systems for agriculture and food applications with nature-derived polymers. iScience 23, 101055. doi: 10.1016/j.isci.2020.101055 32339991 PMC7186528

[B62] Schiavi D. Francesconi S. Taddei A. R. Fortunati E. Balestra G. M. (2022). Exploring cellulose nanocrystals obtained from olive tree wastes as sustainable crop protection tool against bacterial diseases. Sci. Rep. 12, 6149. doi: 10.1038/s41598-022-10225-9 35413981 PMC9005629

[B63] Scortichini M. Loreti S. Pucci N. Scala V. Tatulli G. Verweire D.. (2021). Progress towards sustainable control of *Xylella fastidiosa* subsp. *pauca* in olive groves of salento (Apulia, Italy). Pathogens 10, 668. doi: 10.3390/pathogens10060668 34072394 PMC8228964

[B64] Simoglou K. B. Karataraki A. Roditakis N. E. Roditakis E. (2012). *Euzophera bigella* (Zeller) (Lepidoptera: *Pyralidae*) and *Dasineura oleae* (F. low) (Diptera: *Cecidomyiidae*): emerging olive crop pests in the Mediterranean? J. Pest Sci. 85, 169–177. doi: 10.1007/s10340-012-0418-1

[B65] Technology solutions for future proofing fruit cultivation (2021) Embassy of the netherland. Available at: https://nlintheusa.com/technology-solutions-for-future-proofing-fruit-cultivation/ (Accessed February 23, 2023).

[B66] Tena A. Soto A. Vercher R. F. García-marí F. (2007). Density and structure of *Saissetia oleae* (Hemiptera: *Coccidae*) populations on citrus and olives: relative importance of the two annual generations. Environ. entomology. 36 (4), 700–706. doi: 10.1603/0046-225x(2007)36[700:dasoso]2.0.co;2 17716461

[B67] Tondini E. Petacchi R. (2019). First observations on the parasitoids complex and on the biology of *Dasineura oleae* during an outbreak in Tuscany, Italy. Bull. Insectol. 72, 93–102.

[B68] Torres-Sánchez J. Mesas-Carrascosa F. J. Pérez-Porras F. López-Granados F. (2023). Detection of *Ecballium elaterium* in hedgerow olive orchards using a low-cost uncrewed aerial vehicle and open-source algorithms. Pest Manage. Sci. 79, 645–654. doi: 10.1002/ps.7233 PMC10092466 36223137

[B69] Trichopoulou A. (2022). Olive oil, Greek Mediterranean diet heritage and honoring the past to secure our future: priorities for research and education. Front. Nutr. 9. doi: 10.3389/fnut.2022.1058402 PMC9726726 36505242

[B70] Trkulja V. Tomić A. Iličić R. Nožinić M. Milovanović T. P. (2022). Xylella fastidiosa in Europe: from the introduction to the current status. Plant Pathol. J. 38, 551–571. doi: 10.5423/PPJ.RW.09.2022.0127 36503185 PMC9742796

[B71] Uribeetxebarria A. Arnó J. Escolà A. Martínez-Casasnovas J. A. (2018). Apparent electrical conductivity and multivariate analysis of soil properties to assess soil constraints in orchards affected by previous parcelling. Geoderma 319, 185–193. doi: 10.1016/j.geoderma.2018.01.008

[B72] Velasco-Amo M. D. P. Arias-Giraldo L. F. F. Ecija M. R. de la Fuente L. Marco-Noales E. Moralejo E.. (2022). Complete circularized genome resources of seven strains of *Xylella fastidiosa* subsp. *fastidiosa* using hybrid assembly reveals unknown plasmids. Phytopathology. doi: 10.1094/PHYTO-10-22-0396-A 36441872

[B73] Vincent C. I. Hijaz F. Pierre M. Killiny N. (2022). Systemic uptake of oxytetracycline and streptomycin in huanglongbing-affected citrus groves after foliar application and trunk injection. Antibiotics 11, 1092. doi: 10.1093/jee/toz299 36009961 PMC9405128

[B74] Wang J. H. Che S. C. Qiu L. F. Li G. Shao J. L. Zhong L. (2020). Efficacy of Emamectin Benzoate Trunk Injection Against the Asian Long-Horned Beetle [Anoplophora glabripennis (Coleoptera: Cerambycidae)]. Journal of economic entomology. 113(1), 340–347. doi: 10.1093/jee/toz299 31751456

[B75] Wang L. Zhang J. Peng D. Tian Y. Zhao D. Ni W.. (2022). High-quality genome assembly of *Olea europaea* subsp. *cuspidata* provides insights into its resistance to fungal diseases in the summer rain belt in East Asia. Front. Plant Sci. 13. doi: 10.3389/fpls.2022.879822 PMC9152427 35656016

[B76] Yao W. Guo S. Wang J. Chen C. Yu F. Li X.. (2022). Droplet deposition and pest control efficacy on pine trees from aerial application. Pest Manage. Sci. 78 (8), 3324–3336. doi: 10.1002/ps.6959 35491531

[B77] Zamora M. A. S. Escobar R. F. (2000). Injector-size and the time of application affects uptake of tree trunk-injected solutions. Scientia Hortic. 84, 163–177. doi: 10.1016/S0304-4238(99)00095-3

